# The Early Introduction of Extracorporeal Membrane Oxygenation for Postcardiotomy Cardiogenic Shock Does Not Improve 30-Day Mortality Rates in Low-Volume Centers

**DOI:** 10.7759/cureus.22474

**Published:** 2022-02-21

**Authors:** Shingo Kunioka, Tomonori Shirasaka, Hiroyuki Miyamoto, Keisuke Shibagaki, Yuta Kikuchi, Nobuyuki Akasaka, Hiroyuki Kamiya

**Affiliations:** 1 Department of Cardiac Surgery, Asahikawa Medical University, Asahikawa, JPN; 2 Department of Cardiovascular Surgery, Steel Memorial Muroran Hospital, Muroran, JPN

**Keywords:** low volume centers, mortality, cardiac surgery, extracorporeal membrane oxygenation, postcardiotomy shock

## Abstract

Background and objective

Postcardiotomy cardiogenic shock (PCS) is one of the most critical conditions observed in cardiac surgery. Recently, the early initiation of venoarterial extracorporeal membrane oxygenation (VA-ECMO) has been recommended for PCS patients to ensure end-organ perfusion, especially in high-volume centers. In this study, we investigated the effectiveness of earlier initiation of VA-ECMO for PCS in low-volume centers.

Methods

We retrospectively assessed patients admitted in two of our related facilities from April 2014 to March 2019. The patients who underwent VA-ECMO during peri- or post-cardiac surgery (within 48 hours) were included. We divided the patients into two groups according to the timing of VA-ECMO initiation. In the early initiation of VA-ECMO group, the “early ECMO group,” VA-ECMO was initiated when patients needed high-dose inotropic support with high-dose catecholamines, such as epinephrine, without waiting for PCS recovery. In the late initiation of VA-ECMO group, the “late ECMO group,” VA-ECMO was delayed until PCS was not controlled with high-dose catecholamines, with the intent of avoiding severe bleeding complications.

Results

A total of 30 patients were included in the analysis (early ECMO group/late ECMO group: 19/11 patients). Thirty-day mortality in the entire cohort was 60% (n=18), and there was no significant difference between the two groups (early ECMO group/late ECMO group: 64%/55%, p=0.712). Thirteen and six patients died without being weaned off in the early ECMO (43%) and late ECMO groups (55%), respectively; there was no significant difference between the two groups (p=0.696). The median duration of ECMO support was five days (IQR: 1.5-6.5).

Conclusions

The early initiation of ECMO did not contribute to patients’ 30-day outcomes in low-volume centers. To improve outcomes of ECMO therapy in patients with PCS, centralization of low-volume centers may be required.

## Introduction

Postcardiotomy cardiogenic shock (PCS) is one of the most serious complications of cardiac surgical procedures. The reported incidence of PCS is 3-5% in routine cardiac surgery, and approximately 1% of these patients require postoperative circulatory support due to fatal low cardiac output syndrome (LOS) and being refractory to medical therapy [1-3]. Venoarterial extracorporeal membrane oxygenation (VA-ECMO) has been widely used for cardiopulmonary failure as a short-term mechanical circulatory support [4]. In the past, the VA-ECMO outcomes for PCS were unsatisfactory, with an approximately 70% mortality rate [5], but recent studies in high-volume centers have demonstrated that outcomes could be improved through early initiation of VA-ECMO in patients with PCS [6]. However, experienced staff is needed for ECMO management, and it remains unknown whether an early ECMO initiation strategy would be justified in low-volume centers. The purpose of this study was to compare early versus late initiation of VA-ECMO in patients with PCS with respect to cardiac outcomes in low-volume centers.

## Materials and methods

Patient selection

We retrospectively assessed the data of patients who underwent adult cardiac surgery at two of our related facilities from April 2014 to March 2019. We included adult patients who received VA-ECMO for PCS, refractory to intra-aortic balloon pump or inotropic support peri- or post-cardiac surgery (within 48 hours). PCS was defined as a condition where the patient could not be weaned off cardiopulmonary bypass (CPB) due to left ventricular (LV) failure, right ventricular failure, and biventricular failure and was provided with high-dose inotropic support and/or intra-aortic balloon pump, excluding cardiac tamponades, hypovolemic shocks, and other treatable low blood pressure conditions such as acidemia and septic shock. High-dose inotropic support was defined basically as the use of 5-10 µg/kg/minute of dopamine and dobutamine, more than 0.2 µg/kg/minute of norepinephrine, 0.1 µg/kg/minute of epinephrine, and 1 unit/min of vasopressin, further considering the reactivity for them and the judgment of the anesthesiologist. To minimize heterogeneity, we excluded patients with PCS in whom VA-ECMO was not used.

This study was approved by the ethics committees of both hospitals and performed in accordance with the guidelines laid out in the Declaration of Helsinki (1964). Given the retrospective, observational nature of the study, the requirement of informed consent was waived by the Ethics Committee.

ECMO management

VA-ECMO was initiated when patients could not be weaned off CPB or in patients with LOS early in the postoperative period (within 48 hours). We divided the patients into two groups according to the timing of VA-ECMO initiation. In the early initiation of VA-ECMO group, the “early ECMO group,” VA-ECMO was initiated when the patients required high-dose inotropic support with high-dose catecholamines, without waiting for PCS recovery; thus, they were started on VA-ECMO approximately within 10 minutes. In the later initiation of VA-ECMO group, the “late ECMO group,” VA-ECMO was delayed until PCS was not controlled with high-dose catecholamines, with the intent of avoiding severe bleeding complications.

The cannulation of VA-ECMO is usually from the femoral artery and vein, known as peripheral cannulation, as this enables easy handling of the VA-ECMO lines, allows more rapid extubation, and facilitates decannulation without reopening the chest [7]. Central cannulation is used when it is difficult to cannulate the femoral artery and vein because of vascular stenosis. Both hospital surgeons avoided central cannulation because of the need to reopen the chest to remove the cannulation tube and to mitigate the risk of uncontrollable bleeding. The main endpoint was 30-day mortality.

Statistical analysis

Continuous variables are expressed as medians and IQR. Categorical and sequential variables are expressed as numbers and percentages of patients. The two groups were compared using the Mann-Whitney U test and Fisher’s exact test, as appropriate. A p-value <0.05 was considered statistically significant. Statistical analyses were performed using EZR (Saitama Medical Center, Jichi Medical University, Saitama, Japan), a graphical user interface for R (The R Foundation for Statistical Computing, Vienna, Austria) [8].

## Results

Thirty patients were treated with VA-ECMO for PCS between April 2014 and March 2019. They were divided into two groups: 19 patients in the early ECMO group and 11 in the late ECMO group. Table 1 summarizes the patients’ preoperative characteristics. There were no significant differences in terms of sex, age, BMI, presence of hypertension, diabetes mellitus, hyperlipidemia, chronic kidney disease, hemodialysis support, chronic obstructive pulmonary disease, and measurements of serum creatinine and LV ejection fraction between the two groups.

**Table 1:** Preoperative characteristics ECMO: extracorporeal membrane oxygenation; IQR: interquartile range

Variables	All patients (n=30)	Early ECMO group (n=19)	Late ECMO group (n=11)	P-value
Male sex, n (%)	14 (47)	9 (47)	5 (50)	1.000
Age, years, median (IQR)	74 (69–79)	76 (71–80)	71 (63–76)	0.174
Weight, kg, median (IQR)	57 (50–67)	53 (50–65)	65 (55–80)	0.126
Body mass index, kg/m^2^, median (IQR)	1.5 (1.4–1.7)	1.5 (1.4–1.7)	1.6 (1.5–1.9)	0.444
Hypertension, n (%)	80 (24)	15 (79)	9 (82)	1.000
Diabetes mellitus, n (%)	8 (27)	5 (26)	3 (27)	1.000
Hyperlipidemia, n (%)	10 (33)	7 (37)	3 (27)	0.702
Serum creatinine, mg/dl, median (IQR)	0.90 (0.73–1.21)	0.91 (0.82–1.35)	0.83 (0.64–1.10)	0.355
Chronic kidney disease, n (%)	18 (60)	14 (74)	4 (36)	0.806
Hemodialysis, n (%)	2 (7)	1 (5)	1 (9)	1.000
Chronic obstructive pulmonary disease, n (%)	5 (17)	4 (21)	1 (9)	0.626
Left ventricular ejection fraction				
Good, >60%, n (%)	18 (60)	12 (60)	6 (55)	1.000
Medium, 40–60%, n (%)	7 (23)	4 (21)	3 (27)	1.000
Poor, <40%, n (%)	5 (17)	3 (17)	2 (18)	1.000
Euro 2 score, median (IQR)	5.21 (1.55–7.51)	2.82 (1.52–27.02)	5.03 (1.70–14.40)	0.707

Intraoperative parameters are shown in Table 2. There was no significant difference between the groups in operation procedures (coronary surgery, valve surgery, aortic surgery, and other cardiac surgeries), operation time, CPB time, or aortic cross-clamp time. The operation procedures did not differ between the two groups. However, there was a significant difference between the groups in terms of the duration between the endpoint of CPB and initiation of VA-ECMO. This duration was significantly longer in the late ECMO group (early ECMO group: six minutes; late ECMO group: 60 minutes, p=0.0307).

**Table 2:** Perioperative variables *P<0.05 ACC: aortic cross-clamp; IABP: intra-aortic balloon pumping; CPB: cardiopulmonary bypass; ECMO: extracorporeal membrane oxygenation; IQR: interquartile range; LV: left ventricular

Variables	All patients (n=30)	Early ECMO group (n=19)	Late ECMO group (n=11)	P-value
Operation time, minutes, median (IQR)	548 (356–648)	586 (351–645)	532 (372–655)	0.874
CPB time, minutes, median (IQR)	268 (173–400)	265 (186–397)	275 (133–399)	0.972
ACC time, minutes, median (IQR)	130 (81–217)	141 (97–231)	98 (69–203)	0.717
IABP support, n (%)	17 (57)	12 (63)	5 (45)	0.454
Main Surgery, n (%)				
Aortic	12 (40)	9 (47)	3 (27)	0.442
Valve	16 (53)	9 (47)	7 (64)	0.626
Coronary	14 (47)	9 (47)	5 (46)	1.000
Other	3 (10)	1 (5)	2 (18)	0.537
Duration between CPB and ECMO, minutes, median (IQR)	7 (3.5–31.0)	6 (4.0–8.0)	60 (17.5–142.5)	0.031*
Arterial lactate at the start of ECMO, mmol/L, median (IQR)	79.5 (58.6–96.8)	80.0 (69.5–118.5)	66.0 (54.5–87.5)	0.282
LV venting, n (%)	2 (7)	0 (0)	2 (18)	0.126

Postoperative variables are shown in Table 3. Thirteen patients (68%) were weaned off VA-ECMO because they recovered from PCS in the early ECMO group; however, the mortality rate was as high as 63% (12 patients). On the other hand, six patients (55%) were withdrawn from VA-ECMO because they recovered from PCS in the late ECMO group, and the mortality rate was 55% (six patients). Therefore, there was no significant difference between the groups, and they also required significantly longer VA-ECMO support. The causes of death are also shown in Table 3. In the present study, most deaths were attributed to LOS; however, no patient died due to VA-ECMO-related bleeding complications.

**Table 3:** Postoperative variables *P<0.05 ECMO: extracorporeal membrane oxygenation; LOS: low cardiac output syndrome; IQR: interquartile range

Variables	All patients (n=30)	Early ECMO group (n=19)	Late ECMO group (n=11)	P-value
Mortality, n (%)	18 (60)	12 (63)	6 (55)	0.702
Cause of death, n (%)				
LOS	15 (82)	9 (47)	6 (55)	1.000
Neurological	1 (6)	2 (17)	0 (0)	0.520
Pulmonary	1 (6)	1 (8)	0 (0)	1.000
ECMO weaning off, n (%)	19 (63)	13 (68)	6 (55)	0.696
Mortality after ECMO weaning off, n (%)	8 (42)	6 (78)	2 (33)	1.000
Bleeding, n (%)	19 (63)	10 (53)	9 (82)	0.140
Femoral access, n (%)	24 (60)	16 (84)	8 (73)	0.641
ECMO duration, days, median (IQR)	5 (1.5–6.5)	2 (1–2)	5 (1.5–6.5)	0.037*

## Discussion

The crucial findings of the present study are as follows: (1) the outcome of VA-ECMO in patients with PCS was unsatisfactory, with 60% mortality in our cohort, and (2) there was no significant difference in mortality between the early and late ECMO groups. The results suggested that the early initiation of ECMO for PCS was not superior to late initiation in low-volume centers.

The use of VA-ECMO in patients with PCS has been on the rise; however, there are no guidelines for the management of VA-ECMO for PCS: the decision of when to initiate VA-ECMO for PCS is made according to the individual surgeon’s experience, which is not the commonly followed practice anymore since a multidisciplinary team (MDT) approach to decision-making is implemented currently for the institution of ECMO in the context of refractory PCS. Previous reports have shown that early initiation of VA-ECMO may improve patient outcomes [6,9]. In the present study, there was no significant difference between the early ECMO and late ECMO groups, possibly because our institutes are relatively low-volume centers, including in terms of ECMO weaning-off rates. Biancari et al. suggested that in-hospital mortality was lower in hospitals with greater experience in VA-ECMO for PCS [10]. In their study, they defined “low-volume centers” as centers that had treated fewer than 50 patients with VA-ECMO for PCS within the past eight years. Based on this definition, our institutions can be considered as “low-volume centers.” Saha et al. have reported that the outcomes of VA-ECMO for PCS improved over time [6]. These reports suggest that larger centers for cardiac surgery have advantages in the management of patients with PCS treated with VA-ECMO. Thus, the expansion of cardiac surgery centers may be an important solution for improving the outcomes of VA-ECMO for PCS in terms of both the volume and the expertise that matters.

LV venting is a debatable aspect in the management of VA-ECMO for PCS. Cevasco et al. have suggested that early recognition and aggressive management of LV distension are paramount in caring for the critically ill patient population in the management of VA-ECMO [11]. In the present study, LV venting via the apical approach was performed in only two patients. There are some techniques to achieve LV venting, such as direct cannulation to the LV via the apical approach, using a percutaneous LV assisting device [11]. We did not perform LV venting proactively because of the risk of bleeding complications, and the mentioned technique was not used to manage LV venting partially because of the small number of patients with PCS. Thus, we consider this to be one of the reasons why the early ECMO strategy did not improve patient outcomes in our low-volume-center study. Hence, in the future, it is necessary to consider how to deal with LV venting.

On the other hand, the duration between the cessation of CPB and initiation of VA-ECMO was significantly longer in the late ECMO group. The surgeons may have hesitated to initiate VA-ECMO for PCS because of the potential for bleeding complications associated with using VA-ECMO. This may prompt earlier initiation of VA-ECMO, which could cause less end-organ perfusion. However, in the present study, there was no significant difference in the outcomes between the groups. This result indicates that early ECMO strategy could shorten PCS duration in cases requiring ECMO support, although it was not sufficient to improve the outcomes in low-volume settings.

The major limitation of this study is its retrospective, double-center, non-randomized design, and the small sample size. We paid attention to the time of VA-ECMO initiation for PCS; however, there may have been many other confounding factors, such as intravenous management, respiratory management, nutrition, and surgical elements. Moreover, this study did not investigate whether the patients’ condition was stable in terms of their hemodynamic status; for example, we did not consider the information on inotropic support and volume therapy. To minimize heterogeneity, we excluded patients in whom VA-ECMO for PCS was initiated after 48 hours and controlled for confounding factors, such as septic shock or pulmonary insufficiency. Hence, we investigated only those patients with PCS who were treated with VA-ECMO. A prospective study is required to further investigate the benefits of VA-ECMO for patients with PCS. Also, further research is required to investigate the optimal timing for the initiation of VA-ECMO for PCS.

## Conclusions

Based on our findings, early ECMO initiation could shorten PCS duration; however, it could not improve the outcomes in low-volume settings. Nevertheless, as demonstrated in previous research, early ECMO strategy can improve patient outcomes in high-volume settings. One solution to make early ECMO work in low-volume settings is the centralization of low-volume centers, and this may require devising an effective system for the management of patients with ECMO for PCS in such centers. Moreover, it is important to continue to collect data to improve patient outcomes in low-volume settings.
